# Photosynthetic traits of *Sphagnum* and feather moss species in undrained, drained and rewetted boreal spruce swamp forests

**DOI:** 10.1002/ece3.939

**Published:** 2014-01-17

**Authors:** Laura Kangas, Liisa Maanavilja, Tomáš Hájek, Eija Juurola, Rodney A Chimner, Lauri Mehtätalo, Eeva-Stiina Tuittila

**Affiliations:** 1 Peatland Ecology Group, Department of Forest Sciences, University of HelsinkiP. O. Box 27, Helsinki, FI-00014, Finland; 2 School of Forest Resources and Environmental Science, Michigan Technological University1400 Townsend Drive, Houghton, Michigan, 49931; 3 Institute of Botany, Academy of Sciences of the Czech RepublicDukelská 135, Třeboň, 379 82, Czech Republic; 4 Division of Atmospheric Sciences, Department of Physics, University of HelsinkiP.O. Box 68, Helsinki, FI-00014, Finland; 5 School of Forest Sciences, University of Eastern FinlandP.O. Box 111, Joensuu, FI-80101, Finland; 6 School of Computing, University of Eastern FinlandP.O. Box 111, Joensuu, FI-80101, Finland

**Keywords:** chlorophyll fluorescence, ecophysiology, light responses, peatland, restoration

## Abstract

In restored peatlands, recovery of carbon assimilation by peat-forming plants is a prerequisite for the recovery of ecosystem functioning. Restoration by rewetting may affect moss photosynthesis and respiration directly and/or through species successional turnover. To quantify the importance of the direct effects and the effects mediated by species change in boreal spruce swamp forests, we used a dual approach: (i) we measured successional changes in moss communities at 36 sites (nine undrained, nine drained, 18 rewetted) and (ii) photosynthetic properties of the dominant *Sphagnum* and feather mosses at nine of these sites (three undrained, three drained, three rewetted). Drainage and rewetting affected moss carbon assimilation mainly through species successional turnover. The species differed along a light-adaptation gradient, which separated shade-adapted feather mosses from *Sphagnum* mosses and *Sphagnum girgensohnii* from other *Sphagna*, and a productivity and moisture gradient, which separated *Sphagnum riparium* and *Sphagnum girgensohnii* from the less productive *S. angustifolium*, *S. magellanicum* and *S. russowii*. Undrained and drained sites harbored conservative, low-production species: hummock-*Sphagna* and feather mosses, respectively. Ditch creation and rewetting produced niches for species with opportunistic strategies and high carbon assimilation. The direct effects also caused higher photosynthetic productivity in ditches and in rewetted sites than in undrained and drained main sites.

## Introduction

Approximately 15 million hectares of *Sphagnum* peatlands have been drained to enhance tree growth for forestry, mostly in northern Europe (Joosten and Clarke 2002). Drainage decreases *Sphagnum* cover (Laine et al. 1995; Korpela 2004), leading to cessation of *Sphagnum* biomass accumulation and consequently, to a loss of many ecosystem services that *Sphagnum* provides [e.g., filtration of soluble organic matter and nutrients, carbon store function, and sustenance of species of conservation value (Zak et al. 2011)]. Feather mosses, which have a lower ability to accumulate carbon than *Sphagnum* (Turetsky et al. 2010), show an opposite pattern: their relative cover increases after drainage (Laine et al. 1995; Korpela 2004).

Spruce swamp forests are minerotrophic peatlands with a dense canopy of trees (*Picea abies*). Despite having high biodiversity (Hörnberg et al. 1998) and ecosystem service values when undrained, spruce swamp forests have been among the peatlands most commonly selected for drainage, due to their high productivity when drained for forestry (Joosten and Clarke 2002). In Finland, where drainage for forestry has been most intensive (Päivänen and Hånell 2012), the area of undrained spruce swamp forests has declined from 2 million hectares in the 1950s to 0.8 million hectares (Ilvessalo 1958; Virkkala et al. 2000). The largest decline has occurred in southern Finland, where spruce swamp forests are now classified as a threatened habitat type (Kaakinen et al. 2008). Restoration of spruce swamp forests started in Finland in the 1990s. It is mostly done for nature conservation purposes in protected areas: rewetting is accomplished by blocking the ditches (Aapala and Tukia 2008). Rewetting practices are well developed by now (Aapala and Similä 2013), but ecological restoration success remains to be quantified.

Restoration success can be defined as when the restored site follows a trajectory that leads to pristine-like environmental conditions, and communities and ecosystem functions typical of pristine ecosystems (Dobson et al. 1997). In *Sphagnum* peatlands, such as spruce swamp forests, this includes restoring the growth of *Sphagnum* mosses, which modify the ecosystem physical conditions and are the primary peat-forming plants (van Breemen 1995).

Restoration by rewetting may affect moss carbon assimilation directly, and/or indirectly through change in species composition. The primary direct effect of rewetting on mosses involves a change in moisture: as poikilohydric plants that cannot regulate their water uptake and loss, mosses are dependent on external moisture. *Sphagnum* net photosynthesis is related to its current moisture content that correlates with water table level (Schipperges and Rydin 1998; Strack and Price 2009). Past moisture conditions also affect *Sphagnum* photosynthesis (Schipperges and Rydin 1998). *Sphagnum* mosses are known to grow well in water-saturated conditions (Rochefort et al. 2002), such as those that prevail immediately after successful ditch blocking (Aapala and Tukia 2008). Feather mosses grow better in wet conditions as well, although their abundance is low in wet habitats (Bauer et al. 2007). Feather mosses are normally restricted from water-saturated environments because of physiological constraints and competitive exclusion by *Sphagnum* (Mulligan and Gignac 2001, 2002).

Rewetting may also affect mosses through change in species composition, because species that are specialized to different habitats differ from one another in productivity. For instance, *Sphagnum* species of wet microhabitats show higher growth rates than species of dry microhabitats (Gunnarsson 2005), and feather moss species show lower productivity than *Sphagnum* (Turetsky et al. 2010). Photosynthetic properties differ between plants that are typical to different successional stages: maximum photosynthetic capacity, dark respiration and light compensation point generally decrease from early-to late-successional species (Bazzaz 1979), while the physiological stress experienced by the plants increases (Grime 1977). In peatlands, this development has been described in the succession from early-successional, fast-growing hollow species to drought-and irradiance-stressed hummock species (Granath et al. 2010; Laine et al. 2011b). However, as spruce swamp forests do not have a true hummock-hollow structure and lack the high irradiance that causes stress to mosses in open mires (Hájek et al. 2009), the successional pattern is likely to be different.

Ditches constitute a distinct habitat in drained and rewetted peatlands. In the drained phase, ditches function as a refuge for *Sphagnum* (Laine et al. 1995). Following rewetting, *Sphagnum* biomass in the blocked ditches can help to stabilize site hydrology. Ditches and ditch banks differ from the main site in water table levels, received irradiance and disturbance regime, which may affect moss photosynthetic properties directly or via changes in species composition.

Understanding the mechanisms of *Sphagnum* recovery is vital for understanding the trajectories that lead to peatland restoration success. Thus far, studies on *Sphagnum* growth traits along primary (Laine et al. 2011b) or secondary succession (Granath et al. 2010) have concentrated on unforested open mires. In this study, we focus on the impacts of drainage and rewetting on moss photosynthesis in spruce swamp forests. Measurements on CO_2_ exchange provide information on the photosynthetic efficiency and light responses of the mosses, while chlorophyll fluorescence measures levels of plant stress due to water limitations, light intensity, and/or nutrient supply (Maxwell and Johnson 2000). We expect drainage and rewetting to affect moss carbon assimilation directly and indirectly by changing the moss species composition. Our aim is to quantify the importance of the direct effects and the indirect effects mediated by successional species change.

This study focuses on five parameters: (i) the maximum rate of light-saturated gross photosynthesis (*PMAX*) showing the photosynthetic capacity, (ii) dark respiration (*R*), (iii) light compensation point of net photosynthesis (*PPFD*_c_,): a measure of photosynthetic light-use efficiency at low light, (iv) actual quantum yield of PSII in high light (Φ_PSII_) showing the efficiency of the photosynthetic machinery, and (v) maximum potential quantum yield of PSII (*F*_v_/*F*_m_): a plant stress indicator. Based on ecological knowledge on succession (Grime 1977; Bazzaz 1979) presented above, we expect *Sphagnum* photosynthetic capacity (*PMAX*) to be highest in rewetted sites and in ditches, the early successional habitats; intermediate in undrained sites, the mature habitats; and lowest in drained sites, the suboptimal habitats. Conversely, we expect plants stress levels (measured as decreased *F*_v_/*F*_m_) to be highest in drained, intermediate in undrained and lowest in rewetted sites and in ditches. We expect respiration to be highest in drained and rewetted sites and lowest in undrained sites. We expect the light compensation point (*PPFD*_c_ ,) to be low everywhere but in the ditches, which lack tree cover.

## Methods

We used a dual approach to quantify the impact of drainage and rewetting on mosses. We measured successional changes in moss communities at 36 sites (nine undrained, nine drained, 18 rewetted) and photosynthetic properties of the dominant *Sphagnum* and feather mosses at nine of these sites (three undrained, three drained, and three rewetted).

### Study sites

Originally, before drainage, all sites were similar (*Vaccinium myrtillus* spruce mires, Laine et al. 2012). To enhance tree growth for forestry, ditches were constructed between 1900 and 1980. Drainage had increased tree volume in the sites from undrained levels (Table A1.1 in Appendix 1, *P* = 0.45). Rewetting was conducted between 1995 and 2008 (2001–2003 in the sites sampled for the photosynthesis measurements) by the Finnish state forest agency Metsähallitus by blocking the drainage ditches with peat or wood (Table 1).

**Table 1:** Moss species sampled by site and drainage state.

Site	Drainage state	Year of rewetting (drainage)	Sampled moss species^1^		
			May–August		May^2^
EvLuVK	Undrained	–	*P. schr*		*S. angu*
			*S. girg*		
			*S. mage*		
			*S. wulf*		
SusiLu	Undrained	–	*P. schr*		*S. angu*
			*S. girg*		*S. mage*
			*S. ripa*		
EvLuPa	Undrained	–	*S. russ*		*P. comm*
			*S. girg*		*S. angu*
			*S. mage*		
Ev03ku	Rewetted (via ditch filling)	2003 (1949–1980)	*P. schr*	*S. girg*^3^	*H. sple*
				*S. ripa*^3^	*S. angu*
					*S. russ*
Ev03ma	Rewetted (via ditch filling)	2003 (1949–1980)	*P. schr*	*S. ripa*^3^	*S. angu*
			*S. girg*	*S. russ*^3^	*S. wulf*
		
Ev01VR	Rewetted (via ditch blocking)	2001 (1949–1980)	*P. schr*	*S. ripa*^3^	*S. angu*
			*S. girg*		*S. russ*
					*S. wulf*
LakkOj	Drained	(1949)	*P. schr*		*S. russ*
			*S. girg*		*S. mage*^3^
			*S. mage*		
KoniOj	Drained	(1965)	*P. schr*		*S. angu*
			*S. girg*		*S. russ*
			*S. mage*		
VesiOj	Drained	(1908–1913)	*P. schr*	*S. ripa*^3^	*H. sple*
			*S. girg*		

1 *H. sple *=* Hylocomium splendens, P. schr *=* Pleurozium schreberi, P. comm *=* Polytrichum commune, S. angu *=* Sphagnum angustifolium, S. girg *=* S. girgensohnii, S. mage *=* S. magellanicum, S. ripa *=* S. riparium, S. russ *=* S. russowii S. wulf *=* S. wulfianum*.

2 Additional to the species sampled at all times.

3 Sampled from the ditch.

**Figure A1.1:**
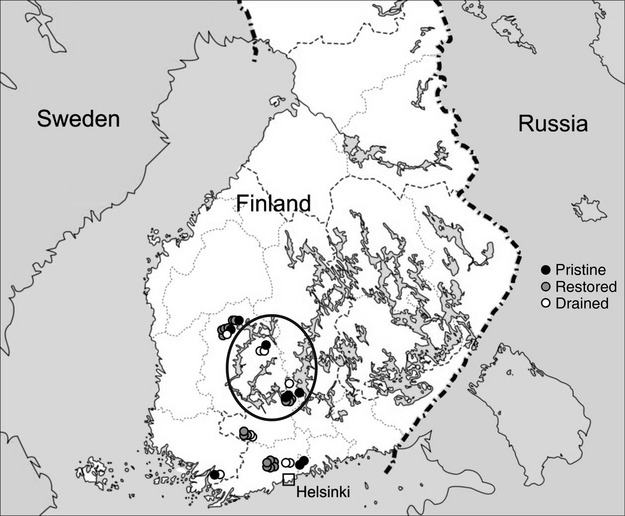
Location of the spruce swamp sites used for the vegetation survey and (circled) for the photosynthesis measurements.

Sites all have peat depths >80 cm and are located in the southern boreal zone, 60−62°N, 23−25°E (for a map, see Appendix 1, Fig. A1.1), at altitudes of 40–170 m a.s.l.. Climate is cold and humid with a long-term mean annual temperature of 3.5–5.3°C and annual precipitation that ranges from 627 to 768 mm depending on location (Table A1.1 in Appendix 1). The average summer 2011 May–August temperature in the sites sampled for the photosynthesis measurements was 14.6°C, which is 1.7°C warmer than the long-term average (1971–2000). Total summer 2011 precipitation was 230 mm, 49 mm less than the long-term average. Norway spruce (*Picea abies*) was the dominant overstorey species at all sites; the understorey was dominated by *Vaccinium* spp. dwarf shrubs.

### Moss cover survey

A vegetation survey was conducted at 36 sites (nine undrained, nine drained, 18 rewetted, see Appendix 1) in 2009. In each site, percent cover of each moss species was estimated in a total of 72 sample plots, 30 cm in diameter, placed in a clustered design. *Sphagnum girgensohnii* and *Sphagnum russowii* were pooled, because they could not be visually identified from each other without extensive effort.

### Photosynthesis measurements

#### Sampling and sample preparation

**Figure 1:**
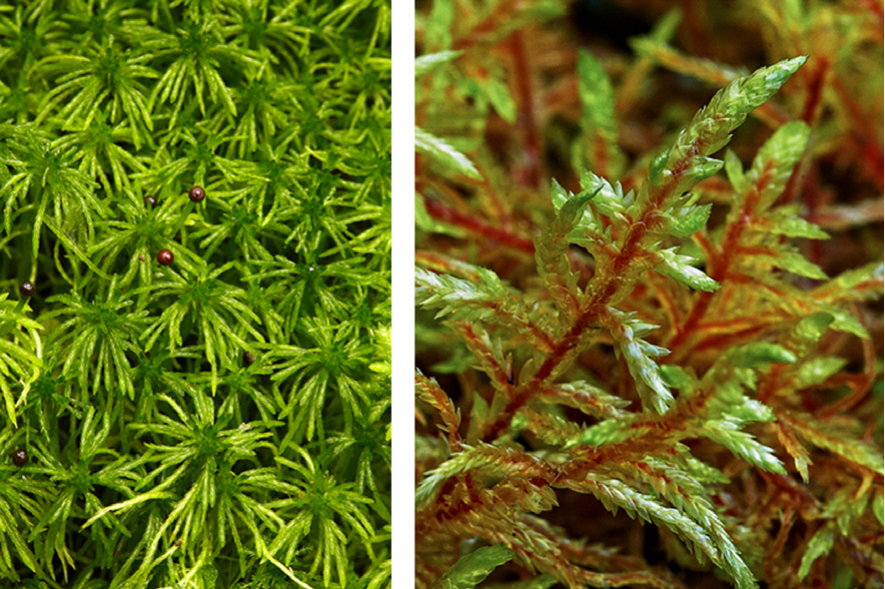
*Sphagnum girgensohnii* (left) and *Pleurozium schreberi* (right) common mosses in undrained and drained spruce swamp forests. Photos: Jukka Laine.

We measured photosynthesis of *Sphagnum* and feather mosses monthly during the summer of 2011. The sampling was designed to account for both the direct and indirect effects of drainage and rewetting: drainage state, variation related to the presence of the ditch habitat and differences between moss species. To eliminate the effect of short-term fluctuation in moisture, the measurements were conducted on acclimatized, moist moss shoots. Dominant moss species in each site (3–4 species, except for the first sample date 4–6) were collected from the most typical habitat for each species (Table 1). *Sphagnum girgensohnii* (Fig. 1, left) and *Pleurozium schreberi* (Fig. 1, right), which were common to all sites, were always collected regardless of dominance. Mosses were collected either from near the ditch (“ditch”) or away from the ditch (“main site”) in the drained and rewetted sites (Table 1).

Three replicates per species were collected each measurement period by cutting the top 5 cm of stems from a 25 cm^2^ area. At each moss collection point, peat moisture of the top 12 cm was measured using a CS-620 HydroSense (Campbell Scientific, Logan, UT) meter. During each sampling period, site water table (WT) was measured manually from three perforated wells that transected the center of each site. In the drained and rewetted sites, one well was located in the ditch line, and two wells transected the main site. Data on tree volume were obtained from tree stand measurements conducted in the sites in 2010. Mosses were stored in polyethylene bags to maintain moisture. After field collection, they were kept in the dark at 5°C for up to 2 days until photosynthesis could be conducted.

#### CO_2_ exchange and chlorophyll fluorescence measurements

CO_2_ exchange and chlorophyll fluorescence were measured in the laboratory using a portable gas exchange fluorescence system GFS-3000 (Heinz Walz GmbH, Effeltrich, Germany). We used a 4 × 2 cm standard chamber that was modified to measure photosynthesis on moss shoot segments. The opaque plexiglass cuvettes (frames) were 1 cm high and equipped with a mesh bottom surface to allow free airflow around the sample. A uniform layer of *Sphagnum* capitula (corresponding to the top 1 cm) was placed in the cuvette. The number of capitula used varied by species and ranged from 5 to 16. For feather mosses, the top 2 cm were cut and placed lengthwise in the cuvette, with stem numbers ranging from 4 to 11. In order to homogenize and minimize water content of the shoots arranged in the cuvettes, we saturated them with drops of water and then blotted them gently from both sides with sheets of pulp until they released no more water. We verified that under these experimental conditions the shoot water content range represented the optimum for CO_2_ exchange.

Prior to measurements, the dark-acclimated samples were allowed to acclimate in the cuvettes for 20 min under a PPFD of 1000 *μ*mol m^−2^ s^−1^ and ambient room temperature of approx. 22°C. Net photosynthesis (*A*) was measured at decreasing levels of PPFD: 1000, 50, 25, and 0 *μ*mol m^−2^ s^−1^ (abbreviated as *A*_1000_*, A*_50_*, A*_25_*,* and *A*_0_) with artificial light provided by a built-in LED light source. *A*_1000_ represents the maximum photosynthetic capacity of the mosses, *A*_50_ to *A*_25_ show net photosynthetic rate in shaded conditions and *A*_0_ represents respiration. Samples were allowed to acclimate to each light level prior to measurement until *A* was constant. During the measurement period, the chamber temperature was kept constant at 20°C, the CO_2_ concentration of incoming air was 400 ppm, air flow was 400 *μ*mol s^−1^ (9.6 mL s^−1^), and the relative humidity of outgoing air was maintained at approximately 90%. Light compensation point of net photosynthesis (*PPFD*_c_) was defined as the level of PPFD where *A *=* *0, calculated from the initial part of the *A*/PPFD curve (from *A*_0_ to *A*_50_)_._ Because *A*_50_ was used for deriving *PPFD*_c_, it was not used as an independent variable in the further data analysis.

Parameters related to photosystem II (PSII) were measured to assess the amount of stress experienced by the mosses, which reflects acclimation of the mosses to their habitats.

Actual quantum yield of PSII photochemistry (Φ_PSII_) and maximum fluorescence (*F*_m_) were measured at the end of the 1000 *μ*mol m^−2^ s^−1^ light level. Samples were then dark acclimated for 6–12 h at 5°C. After the dark acclimation, chlorophyll fluorescence was measured again, and the ratio of variable and maximum fluorescence (*F*_v_*/F*_m_) calculated. The *F*_v_*/F*_m_ ratio represents the maximum potential quantum yield of PSII. After the measurement, samples were dried to a constant weight, and *A* was expressed per unit dry mass (mg g^−1^ h^−1^).

### Data analysis

In the analysis of the effects of drainage and rewetting, we used both classification into drainage states (undrained, drained, rewetted), and classification into habitats (undrained, drained main site, ditch of drained site, rewetted main site, ditch of rewetted site). The latter one acknowledges the marked spatial variation related to the presence of the ditch. In addition, as previous studies have revealed that photosynthetic responses of peatland mosses often vary by season (i.e., Gaberščik and Martinčič 1987), this was taken into account in the analysis.

To quantify the effect of habitat on moss community composition in the 36 sites of the vegetation survey, we used redundancy analysis (RDA) on centered, nontransformed moss species data using the program Canoco 5 (ter Braak and Šmilauer 2012). Statistical significance was evaluated using Monte Carlo permutation restricted for the hierarchical sampling design.

To quantify differences in water table level between the habitats in the nine sites sampled for the measurements, we applied a linear mixed-effects model. In the initial model, habitat, month and the interaction of these two were included as fixed effects. Site was included as a random effect. The interaction was not found significant and was eliminated. Differences in water table level between the habitats and months were compared *post hoc*. We quantified differences in tree stand volume between the drainage states in the nine sites using ANOVA and *post hoc* comparisons. Models were fitted using functions lme and lmer in the lme4 package of R.

To explore the main trends in the variation of the measured photosynthetic response parameters, we used principal component analysis (PCA) on CO_2_ assimilation rate at three levels of PPFD (*A*_1000_, *A*_25_, and *A*_0_), light compensation point of net photosynthesis (*PPFD*_*c*_), actual quantum yield of PSII in high PPFD (Φ_PSII_), and maximum potential quantum yield of PSII (*F*_v_/*F*_m_). The variation in the parameters was projected in relation to habitat, species, peat field moisture, and site water table. As patterns without seasonal variation are easier to interpret, only data from the May measurement period was used for the PCA analysis. This month contained the greatest number of measured species.

To quantify the direct effect of drainage and rewetting against the effect of moss species on moss photosynthetic parameters, we conducted two variation-partitioning analyses: one using the drainage state and another one using the habitat as a predictor variable. The first analysis provides a direct answer to our research question, whereas the second one acknowledges the actual habitat diversity created by drainage and rewetting. We partitioned the variation in the measured photosynthetic response parameters into three components explained by species, month, and drainage state/habitat, testing both simple and conditional effects. This was conducted by creating a partial RDA for each predictor variable with the other predictor variables as covariates. Only the species measured in all 4 months were included in the RDA. CANOCO for Windows 4.5 and 5 (ter Braak and Šmilauer 2002, 2012) was used for the PCA and RDA. The analyses were conducted on centered and standardized photosynthetic parameters as response variables.

To quantify differences between light compensation point of net photosynthesis (*PPFD*_c_), actual quantum yield of PSII (Φ_PSII_), and the maximum potential quantum yield (*F*_v_*/F*_m_), we applied linear mixed-effects models. In the initial models, species, habitat, month, water table, peat field moisture, and sample dry weight were included as fixed predictors. Site was included as a random effect. Fixed effects were eliminated from the model if not found significant (see Table A2.3 in Appendix 2 for the final model results). The differences in *PPFD*_c_, Φ_PSII_*,* and *F*_v_*/F*_m_ between the habitats, species, and months were compared *post hoc* as described previously. Models were fitted using functions lme and gls in the nlme package of R (Pinheiro and Bates 2000).

To determine the effects of habitat and species for photosynthetic capacity, light-use efficiency at low light, and respiration, we applied a nonlinear mixed-effects model with the hyperbolic light saturation curve (i.e., Larcher 2003), within which parameters were linearly dependent on predictor variables:


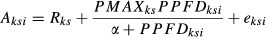
(1)

where the response *A*_*ksi*_ is the observed net photosynthesis and the predictor *PPFD*_*ksi*_ is the photosynthetic photon flux density for measurement *i* of sample *s* on site *k*. The parameters to be estimated are respiration (*R*_*ks*_) photosynthetic capacity i.e. the maximum rate of light-saturated gross photosynthesis (*PMAX*_*ks*_) and the maximum quantum yield of CO_2_ assimilation (*α*). The residual (*e*_*ksi*_) is normally distributed with mean zero and constant variance. Parameter *α* was assumed to be constant over all samples and sites; this restriction was necessary because of the low number of measurements per sample (four PPFD levels with one observation for each). Variation in *R*_*ks*_ and *PMAX*_*ks*_ was explained by the fixed predictors moss species, habitat, month, water level, peat field moisture and sample dry mass, and random effects for site and sample. Final models for the photosynthesis parameters in Eq. 1 are defined below (see Table A2.4 in Appendix 2 for the final model results). All terms in the following models explained the variation in response significantly (approximate *F*-test, *P* < 0.05):


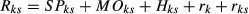
(2)


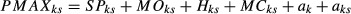
(3)

where *SP*_*ks*_, *MO*_*ks,*_ and *H*_*ks*_ are factor-type predictors for species (9 levels), month (4 levels), and habitat (5 levels), respectively. *MC*_*ks*_ is dry mass of the sample, which has been centralized to have a mean of zero. The last two terms in the equations are random effects for the site and sample, with bivariate normal distributions (*r*_*k*_, *a*_*k*_)' ˜ *MVN*(0, *Σ*_*k*_) and (*r*_*ks*_, *a*_*ks*_)' ˜ *MVN*(0, *Σ*_*ks*_). The random effects account for the correlation arising from the nested grouping of the data to sites and samples within sites. The model was fitted and the tests performed using package nlme of the R software (Pinheiro and Bates 2000).

The differences in *PMAX* and *R* (Eq. 1) between the habitats, species, and months were compared *post hoc*: each habitat was compared against undrained, moss species were compared against *Sphagnum girgensohnii*, and months were compared against July. The difference to undrained shows how drainage and rewetting have changed the photosynthetic parameters from the original natural conditions. Of the moss species, *S. girgensohnii* was chosen as the baseline because it is a common, typical moss species in undrained spruce swamp forests (Laine et al. 2012). July was chosen as the baseline month because it is the usual period of peak growth in the study region (Riutta et al. 2007; Wilson et al. 2007). Significance limit of *P* < 0.05 was used in all analyses.

To test whether the effects of drainage and rewetting differ between *Sphagnum girgensohnii* and *Pleurozium schreberi* – the two species that we sampled in all three drainage states – we conducted a separate test on the interaction effect of species and habitat on Φ_PSII_, *F*_*v*_*/F*_*m*_, *PMAX,* and *R*. Ditch habitats were excluded from the analysis, as *P. schreberi* did not occur in them.

To examine photosynthetic strategies of the moss species *a posteriori*, we classified the species in three categories after Grime (1977): ruderal, competitive, and stress-tolerant, based on their *PPFD*_*c*_ and *PMAX*. The stress-tolerant category was further divided into stress-tolerant, shade species; and stress-tolerant, light species. Ruderal species were defined to show high PPFD_c_ and *PMAX*. Competitive species were defined to show low PPFD_c_, because they are more adapted to the shaded conditions of the spruce swamp forests, and high *PMAX*. Stress-tolerant species were defined to show low *PMAX*. Stress-tolerant shade species were defined to have low PPFD_c_, stress-tolerant light species high PPFD_c_. To test the classification, we ran the models for the photosynthetic parameters using these four groups instead of species.

## Results

### Moss species composition

**Figure 2:**
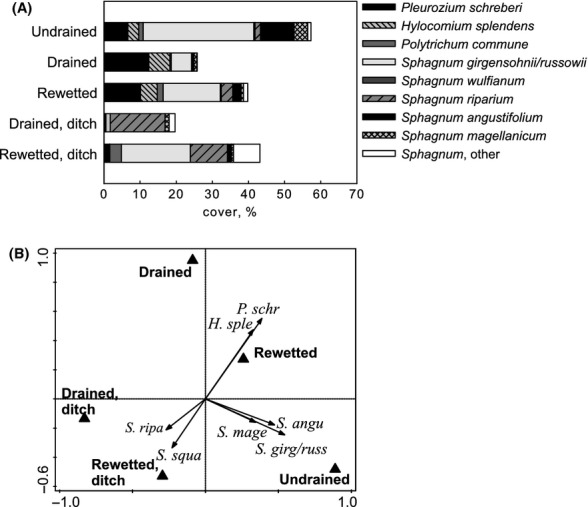
(A) *Sphagnum* and feather moss species cover by habitat; (B) redundancy analysis (RDA) on the effect of habitat on moss community composition. *Sphagnum* and feather moss species with >10% fit shown. First axis explains 15% of the data variation, *P* = 0.004. Second axis explains 7% of the data variation, *P* = 0.002.

Total moss cover was highest in the undrained sites. *S. girgensohnii* coupled with *S. russowii* was favored by undrained conditions, but it was common in all habitats (Fig. 2). *Pleurozium schreberi* and *Hylocomium splendens* were more common in drained and rewetted than in undrained sites. High cover of *Sphagnum riparium* and *S. squarrosum* was typical of ditch habitats, while *S. magellanicum* and *S. angustifolium* were typical species for undrained sites (Fig. 2).

### Photosynthetic properties

#### Environmental conditions

**Figure 3:**
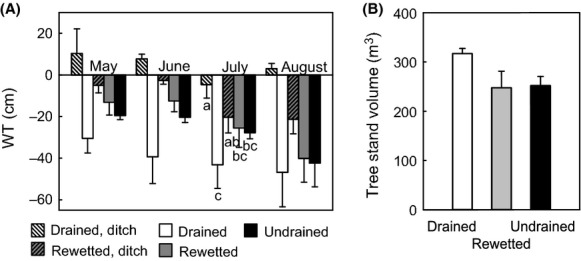
(A) Average water table level (WT) relative to moss surface during the summer season 2011 by habitat and (B) tree stand volume in the study sites by drainage state; Bars indicate SE. Different letters mark significant differences in ANOVA; letters in a) apply to all months, although marked only for July.

In the sites sampled for the photosynthesis measurements, ditches in drained sites had the highest water table, followed by ditches in rewetted sites (Fig. 3A). Rewetted and undrained sites showed similar (*P* = 0.97) water table levels (Fig. 3A). Water tables were lowest in drained sites, but difference to undrained and rewetted sites was not significant (Fig. 3A). Differences in water table between the habitats remained similar over the growing season of 2011, as indicated by a lack of significant interaction effect. Water table levels in May and June were significantly higher than water tables in July and August. Tree stand volume was highest in the drained sites (Fig. 3B), but not significantly so (0.05 < *P*-values<0.10).

#### Main gradients in the data

**Figure 4:**
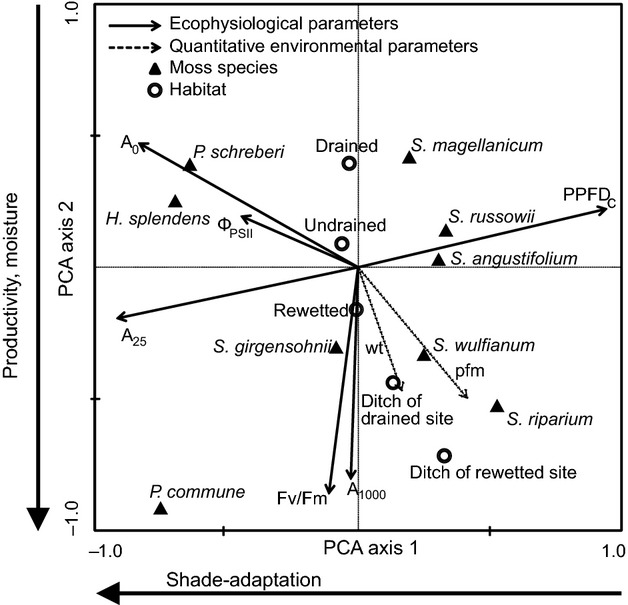
Principal component analysis (PCA) linking photosynthetic response parameters with environmental factors. Data measured during May 2011. Photosynthetic response parameters: CO_2_ assimilation rate at three levels of PPFD (*A*_1000_, *A*_25_, and *A*_0_), light compensation point of net photosynthesis (*PPFD*_*c*_), maximum quantum yield of PSII (*F*_v_/*F*_m_), and quantum yield of PSII (Φ_PSII_). Environmental factors: moss species, habitat, site water table (*wt*), and peat field moisture (*pfm*). Axes 1 (light adaptation) and 2 (productivity and moisture) explain 44% and 30% of total variation, respectively.

Two strong gradients appear in the photosynthetic response data (Fig. 4). The main gradient (PCA Axis 1) is related to photosynthetic efficiency at low light/dark respiration (*A*_0_), photosynthesis at the lowest light level (*A*_25_) and light compensation point of net photosynthesis (PPFD_c_). It separates feather mosses from *Sphagnum* mosses and *S. girgensohnii* from the remaining *Sphagna*. This gradient explains 44% of the variation in photosynthetic properties. The second gradient (PCA Axis 2) is related to productivity and moisture: photosynthesis at high light (*A*_1000_), the stress indicator *F*_v_/*F*_m_, water table and peat field moisture. It reflects differences in both species and habitats. Along this gradient, increased field moisture corresponds to higher productivity and decreased stress. The second gradient explains 30% of the data variation.

#### Species versus habitat influence

Moss species affected variation in the measured photosynthetic properties more than drainage state (Table 2a): species explained 31% of the variation, while drainage state explained 2%. When the presence of ditches is taken into account (Table 2b), combined effect of species and habitat became important, explaining 7% of the data variation: this reflects species differences between ditch habitats and the main sites (Table 1, Fig. 2).

**Table 2:** Variation partioning into fractions explained by moss species, month and (a) drainage state (undrained, drained, rewetted) or (b) habitat (undrained, drained, ditch of drained site, rewetted, ditch of rewetted site). All fractions were significant, *P* < 0.005.

Predictor	% of all variation	Predictor (combined effects)	% of all variation
(a)			
Species	30.5	Species & Month	2.1
Month	16.9	Species & Drainage state	1.7
Drainage state	1.8	Month & Drainage state	<0.1
		Species, Month & Drainage state	−0.1
Total	53.1		
(b)			
Species	25.4	Species & Month	3
Month	17.1	Species & Habitat	6.8
Habitat	3.3	Month & Habitat	−0.1
		Species, Month & Habitat	−1
Total	54.5		

Both species and habitat affected the modeled photosynthetic parameters *PPFD*_*c*_, *PMAX* and *R* significantly (Tables A2.1 and A2.2 in Appendix 2). Also, Φ_*PSII*_ was affected by both species and habitat (Table A2.1). Stress indicator *F*_*v*_*/F*_*m*_ was affected by species, but not habitat: instead, water table level and field moisture were significant predictors for *F*_*v*_*/F*_*m*_ (Table A2.1). Parameter estimates and standard errors for the fixed effects, and standard deviations and correlations for the random effects are presented in Tables A2.3 and A2.4 in Appendix 2.

#### Light adaptation

Low light compensation point of net photosynthesis (*PPFD*_*c*_) is an adaptation to shady environment. PPFD_c_ was lower in undrained than in drained main sites (Table 3a). The lowest PPFD_c_ was found in the ditches of drained sites (Table 3a). Feather mosses *Pleurozium schreberi* and *Hylocomium splendens* had the lowest and *Sphagnum riparium* the highest *PPFD*_*c*_ (Table 3b). *S. wulfianum* and *S. girgensohnii* had lower *PPFD*_*c*_ than the other *Sphagna* (Table 3b).

**Table 3:** Differences in light compensation point (*PPFD*_*c*_), maximum quantum yield of PSII (*F*_*v*_*/F*_*m*_), and quantum yield of PSII (Φ_*PSII*_) between (a) habitat, (b) species (in the order of increasing PPCD_c_), and (c) month. *Post hoc* contrast results from the linear-mixed-effects models. Undrained state, *Sphagnum girgensohnii* and July are the baselines, two of which are kept constant while the predictor variable in question changes. *P*-values indicate significant differences from undrained, *S. girgensohnii*, and July, respectively. Average ± SE, *n *= number of measured moss samples.

	*n*	*PPFD*_*c*_ (*μ*mol m^−2^ s^−1^)	*P*-value^*^	ΦPSII	*P*-value^*^
(a) Habitat *S. girg*, July					
Undrained	49	14 ± 1	–	0.09 ± 0.004	−
Drained, main site	35	16.5 ± 1.19	**0.035**	0.09 ± 0.003	0.596
Rewetted, main site	29	16 ± 1.21	0.096	0.1 ± 0.004	0.145
Drained, ditch	6	9.9 ± 1.95	**0.034**	0.06 ± 0.005	**<0.001**
Rewetted, ditch	19	14.7 ± 1.52	0.655	0.09 ± 0.003	0.838

	*n*	*PPFD*_*c*_ (*μ*mol m^−2^ s^−1^)	*P*-value^*^	ΦPSII	*P*-value^*^	*F*_*v*_*/F*_*m*_	*P*-value
(b) Species Undrained, July							
* Hylocomium splendens*	2	8.83 ± 2.24	**0.022**	0.16 ± 0.02	**0.001**	0.76 ± 0.012	0.7
* Polytrichum commune*	1	11.3 ± 3	0.361	0.16 ± 0.028	**0.023**	0.82 ± 0.016	**<0.001**
* S. wulfianum*	6	13.1 ± 2.2	0.684	0.12 ± 0.007	**<0.001**	0.77 ± 0.007	0.111
* Pleurozium schreberi*	36	14 ± 0.97	0.998	0.17 ± 0.005	**<0.001**	0.75 ± 0.004	**0.001**
* S. girgensohnii*	36	14 ± 1	**−**	0.09 ± 0.004	**−**	0.76 ± 0.007	**−**
* S. magellanicum*	18	18.3 ± 0.96	**<0.001**	0.09 ± 0.003	0.275	0.73 ± 0.005	**<0.001**
* S. angustifolium*	7	19 ± 1.88	**0.009**	0.11 ± 0.007	**0.01**	0.75 ± 0.007	0.109
* S. russowii*	12	20.5 ± 1.27	**<0.001**	0.1 ± 0.004	**0.006**	0.76 ± 0.005	0.481
* S. riparium*	20	22.4 ± 1.24	**<0.001**	0.1 ± 0.003	0.353	0.72 ± 0.006	**<0.001**
(c) Month *S. girg*, Undrained							
May	48	24.1 ± 0.79	**<0.001**	0.12 ± 0.003	**<0.001**	0.738 ± 0.004	**<0.001**
June	30	15.4 ± 0.65	**0.04**	0.1 ± 0.002	0.194	0.775 ± 0.004	**<0.001**
July	30	14 ± 1	**−**	0.09 ± 0.004	**−**	0.76 ± 0.007	−
August	30	14.8 ± 0.64	0.218	0.11 ± 0.003	**<0.001**	0.802 ± 0.004	**<0.001**

Bold font indicates relationship is significant.

#### Productivity

*PMAX* and *R* were higher in ditches and in rewetted sites than in undrained sites, but similar across undrained and drained main sites (Table 4a). Feather mosses, *Pleurozium schreberi* and *Hylocomium splendens,* had the lowest and *Sphagnum riparium* the highest *PMAX*, *R* and net productivity (Table 4b). *S. russowii* and *S. magellanicum* had lower *PMAX* than *S. girgensohnii* but similar *R*, which resulted in lower net maximum productivity than that of *S. girgensohnii* (Table 4b).

**Table 4:** Differences in maximum photosynthetic rate (*PMAX*) and dark respiration (*R*) between; (a) habitat, (b) species (in the order of increasing *PMAX*) and (c) month. *Post hoc* contrast results from the hyperbolic light saturation model (Eq. 1). Undrained state, *Sphagnum girgensohnii* and July are the baselines, two of which are kept constant while the predictor in question changes. *P*-values indicate significant differences from undrained, *S. girgensohnii* and July, respectively. Average ± SE, *n *= number of measured moss samples.

	*n*	*PMAX* (mg g^−1^ h^−1^)	*P*-value^*^	R (mg g^−1^ h^−1^)	*P*-value^*^	*PMAX* + R (mg g^−1 ^h^−1^)
(a) Habitat *S. girg*, July						
Undrained	49	6.73 ± 0.31	−	−0.831 ± 0.075	−	5.9
Drained, main site	35	6.78 ± 0.42	0.902	−0.950 ± 0.051	**0.019**	5.8
Rewetted, main site	6	7.40 ± 0.42	0.113	−1.068 ± 0.092	**0.01**	6.3
Drained, ditch	19	7.71 ± 0.55	0.076	−0.794 ± 0.131	0.778	6.9
Rewetted, ditch	29	7.89 ± 0.47	**0.014**	−1.120 ± 0.107	**0.007**	6.8
(b) Species Undrained, July						
* Pleurozium schreberi*	36	2.68 ± 0.24	**<0.001**	−0.132 ± 0.051	**<0.001**	2.5
* Hylocomium splendens*	2	3.21 ± 0.61	**<0.001**	−0.010 ± 0.159	**<0.001**	3.2
* S. russowii*	12	5.46 ± 0.29	**<0.001**	−0.864 ± 0.075	0.662	4.6
* S. magellanicum*	18	5.55 ± 0.24	**<0.001**	−0.837 ± 0.064	0.923	4.7
* S. angustifolium*	7	6.54 ± 0.34	0.569	−0.996 ± 0.091	0.071	5.5
* S. girgensohnii*	36	6.73 ± 0.31	**−**	−0.831 ± 0.075	**−**	5.9
* Polytrichum commune*	1	7.84 ± 0.83	0.184	−0.923 ± 0.22	0.677	6.9
* S. wulfianum*	6	8.01 ± 0.45	**0.004**	−0.982 ± 0.098	0.125	7
* S. riparium*	20	8.7 ± 0.3	**<0.001**	−1.645 ± 0.078	**<0.001**	7.1

	*n*	R (mg g^−1^ h^−1^)	*P*-value^*^	*PMAX* + R (mg g^−1 ^h^−1^)
(c) Month *S. girg*, Undrained				
May	48	−1.369 ± 0.041	**<0.001**	5.4
June	30	−0.934 ± 0.043	**0.016**	5.8
July	30	−0.831 ± 0.075	**−**	5.9
August	30	−0.886 ± 0.043	0.194	5.8

Bold font indicates relationship is significant.

#### Physiological efficiency and stress

Ditches of drained sites had lower Φ_PSII_ than the other habitats (Table 3a). For *H. splendens*, *P. commune*, and *P. schreberi*, Φ_PSII_ was 50% higher than for *Sphagnum* mosses (Table 3b). *F*_v_*/F*_m_ responded to water level and field moisture, not to habitat (Table A2.1). *F*_v_*/F*_m_ was lowest, i.e. stress was highest, for *S. riparium* and *S. magellanicum*; *Polytrichum commune* showed the highest *F*_v_*/F*_m_ (Table 3b).

#### Seasonality in photosynthetic properties

*PMAX* showed no change across the season (Table 4c), but *R* (Table 4c) and PPFD_c_ (Table 3c) were higher in May and June than in July and August. Plant stress, as indicated by low *F*_v_*/F*_m_, was highest in May and lowest in August (Table 3c).

#### Habitat effect by species

*Sphagnum girgensohnii* and *Pleurozium schreberi* did not differ in their responses to drainage state in most photosynthetic parameters. Only the plant stress indicator *F*_*v*_*/F*_*m*,_ showed a larger difference for the drained state to undrained and rewetted states for *S. girgensohnii* than for *Pleurozium schreberi* (*P* = 0.019). The *F*_*v*_*/F*_*m*_ values (lower values for higher stress) for *P. schreberi* in undrained, drained and rewetted conditions were 0.74, 0.74 and 0.75, respectively; for *S. girgensohnii* 0.76, 0.74 and 0.77.

### Moss strategies

**Figure 5:**
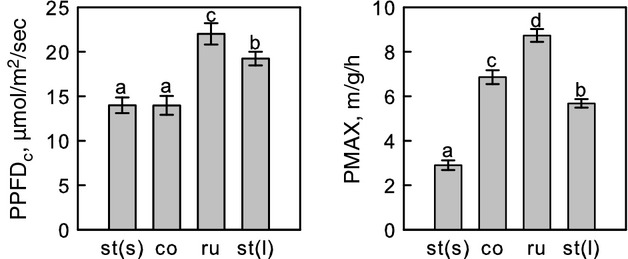
Results from statistical testing on the groups in Table 5, when used in the mixed-effect models as substitutes for species: differences in light compensation point (*PPFD*_*c*_) and maximum photosynthetic rate (*PMAX*). st(s) = stress-tolerant (shade), co = competitive, ru = ruderal, st(l) = stress-tolerant (light). Different letters mark significant differences.

The four groups: stress-tolerant (shade), ruderal, competitive and stress-tolerant (light) (Table 5) functioned as significant predictors in the models for *PPFD*_*c*_ and *PMAX* when used as substitutes for species (see Fig. 5 for the estimates).

**Table 5:** Species classified by their light adaptation, productivity and strategy, based on the photosynthetic response parameters *PPFD*_C_ (light adaptation) and *PMAX* (productivity).

Species	Light adaptation (shade/light)	Productivity (+/−)	Strategy (after Grime 1977)
*Pleurozium schreberi*	Shade	−	Stress-tolerant (shade)
*Hylocomium splendens*	Shade	−	Stress-tolerant (shade)
*Polytrichum commune*	Shade	+	Competitive
*Sphagnum girgensohnii*	Shade	+	Competitive
*S. wulfianum*	Shade	+	Competitive
*S. riparium*	Light	+	Ruderal
*S. magellanicum*	Light	−	Stress-tolerant (light)
*S. russowii*	Light	−	Stress-tolerant (light)
*S. angustifolium*	Light	−	Stress-tolerant (light)

## Discussion

### Spruce swamp forest – favorable habitat for mosses

Minerotrophic, shaded mire environments provide favorable growing conditions for *Sphagnum* mosses (Clymo 1973; Hájek et al. 2009). These conditions are found in rewetted and undrained spruce swamp forests throughout the growing season. Values for *PMAX* were higher than those reported for *Sphagnum* and feather mosses in ombrotrophic bogs (Granath et al. 2009; Laine et al. 2011b), forested permafrost peatlands (Skre and Oechel 1981), a rich fen (Granath et al. 2009) and oligotrophic fens (Laine et al. 2011b). Photosynthetic rates rose gradually from spring to mid-season (July), in contrast to ombrotrophic bogs, where moss growth tends to be greatest in the spring and late summer or autumn (Silvola and Heikkinen 1979; Lindholm 1990; Laine et al. 2011b). A similar gradual rise and mid-season peak in photosynthesis has been measured in a black spruce permafrost peatland in interior Alaska (Skre and Oechel 1981).

Although water table levels were progressively lower toward late summer, values of *F*_v_*/F*_m_ revealed no drought stress to photosystem II values. On the contrary, *F*_v_*/F*_m_ increased toward August. The *F*_v_*/F*_m_ values were higher than those measured in bryophytes from other natural conditions (Hájek et al. 2009; Laine et al. 2011b; Zona et al. 2011), close to values measured in unstressed vascular plants and mosses (Proctor 2010), indicating low levels of light-induced stress. The significant differences we found in *F*_v_*/F*_m_ between habitats, species and species responses to drainage states were too small to be ecologically relevant.

### Direct habitat effects

Although drainage for forestry deteriorates the conditions for *Sphagnum* mosses, as the decreased *Sphagnum* cover indicates, some microsite areas in the drained sites remain suitable for *Sphagnum*: photosynthetic capacity and net production in the mosses of these remnant patches did not differ from undrained conditions. *Sphagnum* mosses were slightly more productive in rewetted than in undrained and drained conditions, but the largest differences occurred between the ditches and other habitats. This direct habitat effect worked to the same direction as the effect mediated by a change in species composition.

### Species responses reflect their growth strategies

Species differences explained the most variation in the photosynthetic response parameters *PPFD*_*c*_*, PMAX,* and *R*. Photosynthetic responses of species reflect their growth strategies. Similarly, to moss species along a primary succession chronosequence of mires (Laine et al. 2011b), responses of the spruce swamp forests moss species reflect environmental gradients in light and moisture. The moss species can be classified in the three groups defined by Grime (1977) as (i) ruderal species that show high production and occupy recently disturbed areas, (ii) competitive species that show high production and occur in more stable conditions, and (iii) stress-tolerant species that show lower production but are more adapted to stress or resource scarcity (Table 5).

*Sphagnum riparium* is most commonly found at the surface water level (Gignac et al. 1991) and is frequently a pioneer species in peatlands that experience a rise in water table level (Zoltai 1993). *S. riparium* displayed characteristics of ruderal vascular plants (Grime 1977; Bazzaz 1979), with high net productivity*, PMAX*, *R,* and *PPFD*_c_.

*Sphagnum girgensohnii* is the dominant moss species in rewetted sites and appears to be competively superior. It had the highest net photosynthesis of all species in rewetted sites outside the ditch line. Previous research indicates *S. girgensohnii* to be an opportunist species in new habitats and a key driver of paludification of boreal maritime forests in North America (Noble et al. 1984; Asada et al. 2004). Similar factors, disturbances to the forest floor together with increased water table, contributed to the increased dominance of *S. girgensohnii* in those forests and in our rewetted sites. Values of *F*_v_/*F*_m_ were always high, except for a slight decline in drained sites: the low stress level indicates fairly large ecological amplitude for this species. *Sphagnum girgensohnii* also differed from the remaining *Sphagnum* mosses by its lower light compensation point, which indicates suitability to the shaded habitat of spruce swamp forests.

Feather mosses *P*. *schreberi* and *H. splendens* had low carbon assimilation and dark respiration rates and low light compensation points. They could be classified as stress-tolerant species, as they are adapted to shaded, dry forest conditions. Another group of stress-tolerants is the hummock-*Sphagna*: *S. magellanicum, S. russowii,* and *S. angustifolium*. They are not specifically adapted to the shaded conditions of spruce swamp forests but tolerate drought by forming tight cushions (Clymo 1973).

### Change in ecosystem photosynthetic traits through succession

Species turnover along the sequence of changed conditions – drainage and ditch creation, rewetting, and development of pristine-like conditions – affects photosynthetic properties of the spruce swamp forest ecosystem. Both undrained and drained spruce swamp forests can be compared with the late-successional stage of forested vascular plant communities, where succession is associated with decreased availability of resources (Grime 1977). Hummock-*Sphagna* (*S. magellanicum*, *S. russowii,* and *S. angustifolium*) are typical species of the undrained late-successional stage, while feather mosses (*Pleurozium schreberi* and *Hylocomium splendens*) are typical of the drained late-successional stage. Ditch creation and rewetting are disturbances that create niches for species with opportunistic strategies. In our study, main PCA gradients separated the drier, more stabilized undrained and drained sites from the wet and disturbed rewetted sites and ditch habitats. The three species strategies, as defined by Grime (1977), can be placed along the successional gradient: stress-tolerant *P. schreberi, S. magellanicum*, *S. russowii,* and *S. angustifolium* at the late-successional stages, ruderal *S. riparium* occupying recently disturbed areas and competitive *S. girgensohnii* during mid-succession.

Ditches of drained sites offered a suitable refuge for *Sphagnum* species to persist. In the rewetted sites, ditches are habitats of highly productive *Sphagnum* cover, primarily *S. riparium*. Over time, the high rate of production of the ruderal *S. riparium* will accelerate terrestrialization of the ditch line, which will lower the relative water table and create suitable microhabitat for other species.

### Implications

Functional trait analysis is a useful method for assessing the outcome of ecological restoration (Hedberg et al. 2013), but the established traits, developed for vascular plants, do not reflect bryophyte ecology or performance (Rice et al. 2008). Photosynthetic properties of mosses are directly linked to their evolutionary strategies. If they are species-specific, as we here show, they can be used as traits in functional trait analysis when coupled with plant cover estimations. Light compensation point for net photosynthesis (*PPFD*_*c*_), and maximum photosynthesis (*PMAX)* appeared useful in understanding the functional variation in spruce swamp forest mosses.

Peatland restoration monitoring commonly depends on comparing restored to pristine sites, which implies straightforward directional change. This can be justified in ombrotrophic bogs, where vegetation changes after drainage and rewetting can be small, because few species are able to live in such acid and nutrient-poor conditions (Laine et al. 2011a). In minerotrophic peatlands, development after rewetting involves more species turnover along the successional trajectory (Haapalehto et al. 2011; Hedberg et al. 2012). Although the species and trait composition of the rewetted sites differs from undrained systems, especially in the blocked ditches, the ruderal and competitive species are likely to contribute to the rapid biomass production and peat formation during the initial stages after rewetting. Later, the ruderal species are likely to become outcompeted by other species of *Sphagnum*. Measurement-based information on species functional traits along successional trajectories enables restoration monitoring to identify different stages of restoration succession.

## Appendix

### Appendix 1

**Table A1.1:** Spruce swamp sites used for the vegetation survey and (colored) for the photosynthesis measurements: time of drainage and rewetting, location as coordinates (EUREF, ˜WGS84), mean annual temperature and annual precipitation in the nearest weather station (1971–2000, Finnish Meteorological Institute), average water table depth (WT) as centimeters below moss surface (from manual measurements in July–August 2009, May-June 2010, May 2011, September 2011, and May 2012), tree stand volume and *Sphagnum* cover.

Code	Drainage state	Year of drainage	Year of rewetting	*N*-coordinate	Mean annual temperature (°C)	Annual precipitation (mm)	Average WT (cm)	Tree stand volume (m^3^)	*Sphagnum* cover (%)
SiLuE	Undrained	–	–	6,686,952	5.3	682	−13	128	62
SiLuW	Undrained	–	–	6,686,925	5.3	682	−18	367	13
TeLu	Undrained	–	–	6,683,434	5.7	768	−28	216	26
RuOjSP	Drained	1932	–	6,692,132	5.3	682	−33	553	6
RuOjSu	Drained	1926	–	6,693,212	5.3	682	−72	329	0
TeOj	Drained	?	–	6,684,069	5.7	768	−39	169	23
Nu97	Rewetted	1960s	1997	6,689,606	4.6	647	−16	244	32
Nu01hi	Rewetted	1960s	2001	6,687,472	4.6	647	−19	356	41
Nu01W	Rewetted	1960s	2001	6,687,779	4.6	647	−6	133	27
Nu05ku	Rewetted	1960s	2005	6,689,992	4.6	647	−30	218	2
Nu05ma	Rewetted	1960s	2005	6,689,683	4.6	647	−5	237	11
Nu08Po	Rewetted	1960s	2008	6,686,957	4.6	647	−13	319	10
AmLu	Undrained	–	–	6,799,071	4.2	645	−27	248	35
EvLuPa	Undrained	–	–	6,792,386	4.2	645	−28	217	56
EvLuVK	Undrained	–	–	6,791,370	4.2	645	−23	280	47
LiOjN	Drained	?	–	6,729,922	4.6	627	−50	320	1
LiOjS	Drained	?	–	6,729,259	4.6	627	−53	403	5
VesiOj	Drained	1908–1913	–	6,806,413	4.6	627	−47	319	3
Li95So	Rewetted	1930s	1995	6,730,416	4.6	627	−7	29	66
Li98	Rewetted	?	1998	6,728,127	4.6	627	−44	311	2
Li00	Rewetted	?	2000	6,733,287	4.6	627	−4	61	21
Ev01VR	Rewetted	1949–1980	2001	6,790,027	4.2	645	−10	181	25
Ev03ku	Rewetted	1949–1980	2003	6,789,004	4.2	645	−32	287	8
Ev03ma	Rewetted	1949–1980	2003	6,788,229	4.2	645	−7	275	33
SusiLu	Undrained	–	–	6,861,522	3.5	711	−16	259	69
HeLu	Undrained	–	–	6,884,392	3.5	711	−25	278	56
SeLu	Undrained	–	–	6,869,326	3.5	711	−15	192	54
LakkOj	Drained	1928;1949	–	6,854,767	3.5	711	−27	334	5
KoniOj	Drained	1965	–	6,854,362	3.5	711	−43	300	19
SeOj	Drained	?	–	6,867,509	3.5	711	−38	263	11
Se95M	Rewetted	1930–1963	1995	6,869,355	3.5	711	−16	268	41
Se96K	Rewetted	1900–1925	1996	6,869,038	3.5	711	−29	289	19
Se98	Rewetted	1963–1976	1998	6,868,705	3.5	711	−23	341	16
He00	Rewetted	1960s–70s	2000	6,879,392	3.5	711	−11	224	39
Se04	Rewetted	1900–1925	2004	6,874,078	3.5	711	−6	275	28
He08	Rewetted	1960s–70s	2008	6,880,730	3.5	711	−25	227	37

## Appendix

### Appendix 2: Statistical models

**Table A2.1:** ANOVA results of the linear mixed–effects models for the differences in light compensation point (*PPFD*_*c*_), actual quantum yield of PSII in high light (Φ_*PSII*_) and maximum potential quantum yield of PSII (*F*_*v*_*/F*_*m*_). WT denotes water table level, VWC peat volumetric water content.

Source	*PPFD*_*c*_				Φ_PSII_				*F*_v_/*F*_m_			
	num. df	den. df	*F*−value	*P*-value	num. df	den. df	*F*-value	*P*-value	num. df	den. df	*F*-value	*P*-value
Intercept	1	387	1331	<0.001	1	387	50157	<0.001	1	391	13618	<0.001
Species	8	387	69	<0.001	8	387	13	<0.001	8	391	15	<0.001
Month	3	387	70	<0.001	3	387	70	<0.001	3	391	80	<0.001
Habitat	4	387	6	<0.001	4	387	6	<0.001				
WT					1	387	8	0.005	1	391	21	<0.001
VWC					1	387	6	0.017	1	391	6	0.012
Dry mass	1	387	46	<0.001								

**Table A2.2:** ANOVA results of the hyperbolic light saturation model (Eq. 1) for the differences in maximum photosynthesis (*PMAX*) and dark respiration (*R*).

	num. df	den. df	*F*-value	*P*-value	num. df	den. df	*F*-value	*P*-value
*α*								
Constant	1	1206	3223	<0.0001				

*PMAX*					*R*				
Intercept	1	1206	464	<0.0001	Intercept	1	1206	123	<0.0001
Species	8	1206	49	<0.0001	Species	8	1206	56	<0.0001
Habitat	4	1206	2	0.049	Month	3	1206	73	<0.0001
Dry mass	1	1206	162	<0.0001	Habitat	4	1206	3	0.022

**Table A2.3:** Parameter estimates of the linear mixed-effects models for the differences in light compensation point (*PPFD*_*c*_), actual quantum yield of PSII in high light (Φ_*PSII*_) and maximum potential quantum yield of PSII (*F*_*v*_*/F*_*m*_).

	*PPFD*_c_ (*μ*mol m^−2 ^s^−1^)			Φ_PSII_			*F*_*v*_*/F*_*m*_		
	Coeff.	SE	*P*-value	Coeff.	SE	*P*-value	Coeff.	SE	*P*-value
Fixed part									
Constant (*S. g*. Jul. Prist.)	14.01	0.99	0.000	0.093	0.004	0.000	0.760	0.007	0.000
* Hylocomium splendens*	−5.18	2.24	0.022	0.063	0.020	0.001	−0.005	0.012	0.700
* Polytrichum commune*	−2.74	2.99	0.361	0.065	0.028	0.023	0.063	0.016	0.000
* S. wulfianum*	−0.90	2.21	0.684	0.026	0.007	0.000	0.012	0.007	0.111
* Pleurozium schreberi*	−0.00	0.97	0.998	0.080	0.005	0.000	−0.014	0.004	0.001
* S. magellanicum*	4.31	0.96	0.000	−0.003	0.003	0.275	−0.029	0.005	0.000
* S. angustifolium*	4.95	1.88	0.009	0.018	0.007	0.010	−0.011	0.007	0.109
* S. russowii*	6.45	1.28	0.000	0.011	0.004	0.006	−0.003	0.005	0.481
* S. riparium*	8.35	1.24	0.000	0.003	0.003	0.353	−0.037	0.006	0.000
Drained, ditch	−4.16	1.95	0.034	−0.029	0.005	0.000			
Rewetted, ditch	0.68	1.52	0.655	0.001	0.003	0.838			
Rewetted, main site	2.03	1.22	0.096	0.005	0.004	0.145			
Drained, main site	2.53	1.19	0.035	−0.001	0.003	0.596			
May	10.1	0.79	0.000	0.029	0.003	0.000	−0.022	0.004	0.000
June	1.34	0.65	0.040	0.003	0.002	0.194	0.015	0.004	0.000
August	0.80	0.65	0.218	0.016	0.003	0.000	0.042	0.004	0.000
Water table				0.000	0.000	0.000	0.00049	0.00011	0.000
VWC							0.00018	0.00007	0.012
Dry mass	0.11	0.016	0.000						
Random part									
sd (constant|site)	1.24			−			0.0107		
Residual standard error	0.94^*^PPFD_C_^0.704			1.04^*^Φ_PSII_^1.78			0.0264		

**Table A2.4:** Parameter estimates of the hyperbolic light saturation model (Eq.1) based on nonlinear mixed-effects model fit.

Fixed part	Coeff.	SE	*P*-value
α			
Constant	73.515	1.294	0.000
*PMAX* (mg^−1^ h^−1^)			
Constant (*S. girgensohnii*, July, Undrained)	6.733	0.313	0.000
* Pleurozium schreberi*	−4.052	0.235	0.000
* Polytrichum commune*	1.104	0.831	0.184
* S. angustifolium*	−0.195	0.342	0.569
* S. magellanicum*	−1.186	0.244	0.000
* S. riparium*	1.967	0.302	0.000
* S. russowii*	−1.275	0.286	0.000
* S. wulfianum*	1.273	0.447	0.004
* Hylocomium splendens*	−3.526	0.610	0.000
Drained, ditch	0.979	0.550	0.076
Rewetted, main site	0.669	0.422	0.113
Drained, main site	0.051	0.416	0.902
Rewetted, ditch	1.161	0.473	0.014
Dry mass	−0.037	0.00288	0.000
*R* (mg g^−1^ h^−1^)			
Constant (*S. girgensohnii*. July, Undrained)	−0.831	0.075	0.000
* Pleurozium schreberi*	0.699	0.051	0.000
* Polytrichum commune*	−0.092	0.220	0.677
* S. angustifolium*	−0.165	0.091	0.071
* S. magellanicum*	−0.006	0.064	0.923
* S. riparium*	−0.814	0.078	0.000
* S. russowii*	−0.033	0.075	0.662
* S. wulfianum*	−0.151	0.098	0.125
* Hylocomium splendens*	0.821	0.159	0.000
June	−0.103	0.043	0.016
August	−0.055	0.043	0.194
May	−0.538	0.041	0.000
Drained, ditch	0.037	0.131	0.778
Rewetted, main site	−0.237	0.092	0.010
Drained, main site	−0.119	0.090	0.019
Rewetted, ditch	−0.289	0.107	0.007

Random part	Site	Sample in site
sd(*PMAX*)	0.451	1.19
sd(*R*)	0.092	0.11
corr(*PMAX*^*^*R*)	−0.843	−0.998
Residual standard error	0.444	
